# Protective Effects of Flavone from *Tamarix aphylla* against CCl_4_-Induced Liver Injury in Mice Mediated by Suppression of Oxidative Stress, Apoptosis and Angiogenesis

**DOI:** 10.3390/ijms20205215

**Published:** 2019-10-21

**Authors:** Bishoy El-Aarag, Asmaa Khairy, Shaden A. M. Khalifa, Hesham R. El-Seedi

**Affiliations:** 1 Biochemistry Division, Chemistry Department, Faculty of Science, Menoufia University, Shebin El-Koom 32512, Egypt; 2 Division of Chemistry and Biotechnology, Graduate School of Natural Science and Technology, Okayama University, Okayama 7008530, Japan; 3 Chemistry Department, Faculty of Science, Menoufia University, Shebin El-Koom 32512, Egypt; asmaakhairybio@gmail.com; 4 Department of Molecular Biosciences, The Wenner-Gren Institute, Stockholm University, SE 106 91 Stockholm, Sweden; shaden.khalifa@su.se; 5 Department of Experimental Cancer Medicine (ECM); Novum, 14157 Huddinge, Sweden; 6 Division of Pharmacognosy, Department of Medicinal Chemistry, Uppsala University, Biomedical Centre, Box 574, SE-75123 Uppsala, Sweden; 7 International Research Center for Food Nutrition and Safety, Jiangsu University, Zhenjiang 212013, China; 8 Al-Rayan Research and Innovation Center, Al-Rayan Colleges, Medina 42541, Saudi Arabia

**Keywords:** liver injury, CCl_4_, *Tamarix aphylla*, oxidative stress, apoptosis, angiogenesis

## Abstract

The current study aimed to investigate, for the first time, the beneficial effects of 3,5-dihydroxy-4′,7-dimethoxyflavone isolated from *Tamarix aphylla* L. against liver injury in mice. Liver injury was induced by intraperitoneal (i.p.) injection of carbon tetrachloride (CCl_4_) at a dose of 0.4 mL/kg mixed in olive oil at ratio (1:4) twice a week for 6 consecutive weeks. The administration of CCl_4_ caused significant histopathological changes in liver tissues while the pre-treatment with the flavone at dose of 10 and 25 mg/kg ameliorated the observed liver damages. Also, it markedly reduced hepatic malondialdehyde (MDA) level as well as increased the activities of liver superoxide dismutase (SOD), catalase (CAT), and glutathione peroxidase (Gpx) compared with their recorded levels in CCl_4_ model group. Moreover, the immunohistochemical analysis demonstrated the enhancement in the protein level of B-cell lymphoma-2 (Bcl-2) while the protein levels of cysteine-aspartic acid protease-3 (caspase-3), Bcl-2-associated x protein (Bax), transforming growth factor-β1 (TGF-β1) and CD31 were suppressed following the flavone treatement. These results suggest that the flavone can inhibit liver injury induced in mice owning to its impact on the oxidation, apoptotic and angiogenesis mechanisms. Further pharmacological investigations are essential to determine the effectiveness of the flavone in human.

## 1. Introduction

The liver plays an essential role in some of the vital body functions including protein synthesis, detoxification, and the metabolism of various consumed or absorbed substances [1,2,3]. Hence, the liver is the detoxification factory and the most exposed to the ingested and injected chemicals and drugs [1]; therefore, the excessive exposure to toxic or hazardous substances could induce liver injury on the long run, affecting the life quality and health status [4]. Carbon tetrachloride (CCl_4_), is a solvent, and a potent hepatotoxic agent that is widely used in animal models for induction of liver damage [5]. Its mechanism of action started at the hepatocytes where the activation of cytochrome P_450_ enzyme produces trichloromethyl and trichloromethyl peroxide free radicals. These free radicals tightly bind to the phospholipid molecules of the cell membranes, endoplasmic reticulum, and mitochondria, causing lipid peroxidation, oxidative stress and the release of byproducts that block the intracellular proteins and DNA [6,7]. The reactive aldehydes that produced as byproducts of lipid peroxidation subsequently damage the structure and function of the cellular and intercellular membranes, resulting in hepatotoxicity and carcinogenicity [8].

Superoxide dismutase (SOD) possess an antioxidant effect against superoxide radicals in cells [9]. Catalase (CAT) neutralizes the harmful effects of peroxide radicals in biological systems [10]. Glutathione peroxidase (GPx) is an active scavenger of peroxides in cells [11]. Oxidative stress-induced tissue damage is defined as the imbalance between oxidation and anti-oxidation generating burdened free radicals and reducing the anti-oxidant capacity [12]. Apoptosis, similarly is the programmed cell death that could be attributed to oxidative stress and accumulation of free radicals. Apoptosis is the main cell death mechanism recognized following the induction of CCl_4_ liver injury [13,14]. Consequently, anti-oxidant and anti-apoptosis therapies exhibited desirable effects for prevention or treatment of liver diseases in animal models [15,16].

Flavonoids are secondary plant metabolites responsible for colour and aroma of flowers. They interfere with multiple signal transduction pathways involved in liver diseases leading to inhibition of oxidative stress, apoptosis, and angiogenesis [17]. A natural flavonoid puerarin attenuated CCl_4_-induced hepatotoxicity by reducing ROS production, restoring the antioxidant enzyme system and regulating hepatic lipid biosynthesis level [18]. Also, quercetin protected liver from CCl_4_-induced injury via antioxidative stress and anti-inflammation through inhibition of Toll-like receptor 2 (TLR2) and Toll-like receptor 4 (TLR4) activations and mitogen-activated protein kinase (MAPK) phosphorylation [19]. A marine polyphenol, dieckol, mediated apoptosis-regulating genes via downregulation of Bax and upregulation of Bcl-xl protein expressions [20]. Eupafolin blocked vascular endothelial growth factor (VEGF) in human umbilical vascular endothelial cells and inhibited Akt activity and VEGF secretion in HepG2 [21]. Morusin, inhibited angiogenesis via attenuation of the IL-6 and activation of transcription 3 (STAT3) signalling pathway in mice bearing HepG2 cells [22]. Resveratrol exhibited effects on suppressing angiogenesis in mice with hepatocellular carcinoma (HCC) xenograft through inhibiting VEGF expression by a NF-*κ*B-mediated pathway [23].

**Figure 1:**
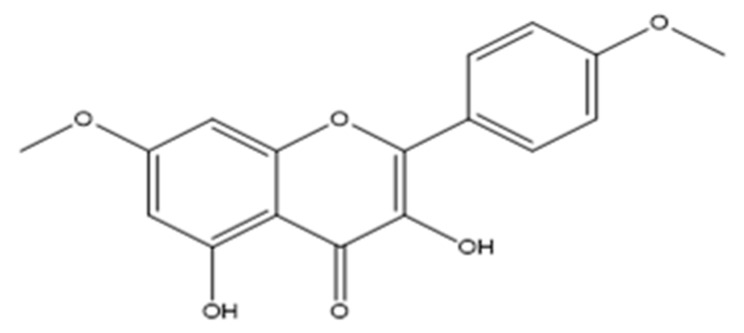
Chemical structure of 3,5-dihydroxy-4′,7-dimethoxyflavone.

*Tamarix aphylla* L. is an evergreen tree belongs to tamaricaceae family that is distributed worldwide [24,25,26]. T. *aphylla* possessed anti-oxidant activity [27] owned to its content of different flavonoids [28] and phenolics [29] with potential effects in prevention and treatment of many diseases [30,31,32]. As part of our ongoing research to identify a new effective and functional natural component [33,34,35,36] with high availability and low cost, the present study aims to investigate the anti-oxidant, anti-apoptotic, and anti-proliferative activities of 3,5-dihydroxy-4′,7-dimethoxyflavone (Figure 1) isolated from *T. aphylla.* The long-term goal is to develop a potent pharmaceutical agent that inhibits the production and activation of free radicals and acts against CCl_4_-induced liver injury in mice.

## 2. Results

### 2.1. Chemical Elucidation of the Flavone

The structure of the compound was established by chemical and spectral analysis, mainly MS, UV and ^1^H-NMR. 3,5-Dihydroxy-7,4′-dimethoxyflavone: Yellow, amorphous powder; UV (MeOH) λmax nm: 211, 233, 269, 327, 368; IR (KBr) νmax 3314, 2922, 2848, 1836, 1743, 1657, 1596, 1507, 1463, 1355, 1318, 1258, 1220, 1162, 1033 cm^-1^; ^1^H-NMR (CDCl_3_, 300 MHz): δ 11.71 (1H, s, H-O-5), 8.14 (2H, d, J= 9.0 Hz, H-2′ and H-6′), 7.01 (2H, d, J= 9.0 Hz, H-3′ and H-5′), 6.58 (1H, s, H-O-3), 6.46 (1H, d, J= 2.1 Hz, H-8), 6.35 (1H, d, J= 2.1 Hz, H-6), 3.87 (3H, s, H3CO-7), 3.86 (3H, s, H3CO-4′). ^13^C-NMR (CD3OD, 300 MHz): δ 175.2 (C-4), 165.7 (C-7), 161.1 (C-4′), 160.8 (C-5), 156.8 (C-9), 145.7 (C-2), 135.7 (C-3), 129.4 (C-2′ and C-6′), 123.2 (C-1′), 114.1 (C-3′ and C-5′), 103.9 (C-10), 97.9 (C-6), 92.2 (C-8), 55.8 (CH_3_O-7), 55.4 (CH_3_O-4′). HREI-MS: m/z 314.078 calculated for C_17_ H_14_ O_6_ (Calcd. 314.079).

### 2.2. Histopathological Analysis of the Liver Tissues

**Figure 2:**
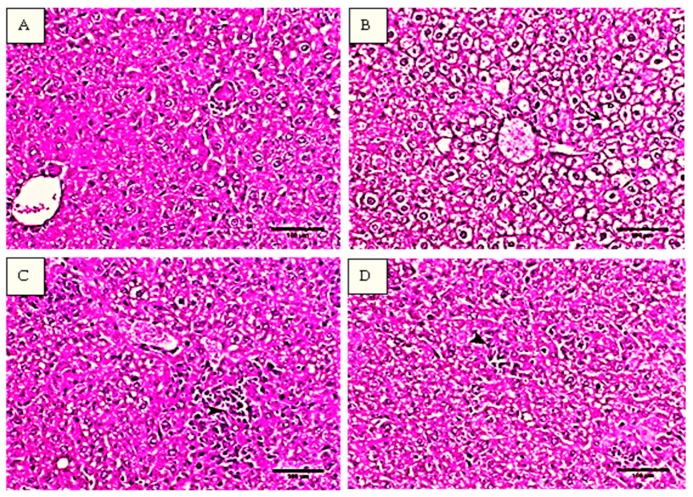
Histopathological graphs of liver sections stained by hematoxylin and eosin (H&E). (**A**): control group, showing normal hepatocytes with oval cytoplasm and with vesicular-shaped nucleus. (**B**): CCl_4_ model group, arrowhead presented multifocal granuloma like lesions. (**C**): CCl_4_ + flavone (10 mg/kg) group, illustrated marked decrease the number of focal infiltrative areas and with remarkable decrease the number of neoplastic cells (arrowhead). (**D**): CCl_4_ + flavone (25 mg/kg) group, arrowhead revealed decrease hepatic degeneration, mild degree of cell swelling, and small number of mononuclear inflammatory cells. Scale bar = 100 µm.

Histopathological analysis of the liver tissues from the studied groups was illustrated in Figure 2. In Figure 2A, the histopathological examination of the liver tissues of normal control mice showed normal hepatocytes arranged in cords around the central vein and separated with blood sinusoids. The hepatocytes have oval cytoplasm and vesicular-shaped nucleus. On the other hand, the liver tissues of mice treated with CCl_4_ showed multiple histopathological changes manifested by the infiltration of mononuclear inflammatory cells mainly macrophage and lymphocytes mixed with multiple neoplastic cells and seen as multifocal granuloma like lesions within the whole hepatic parenchyma similar to ehrlich ascites carcinoma cells (EACs). The infiltrative inflammatory cells were also noticed in periportal area and within blood sinusoids. The findings as illustrated in Figure 2B showed swollen hepatocytes with diffuse vacuolation and granular disrupted cytoplasm.

The pretreatment with flavone (10 mg/kg) protected the hepatocytes and remarkable decrease in the number of focal infiltrative cells in particular neoplastic cells was demonstrated. The congested areas showed mostly an increase in the necrobiotic changes and migration of the histocytes (Figure 2C). However, the vacuolation was still noticed within the hepatic parenchyma as the hepatocytes showed presence of clear round cytoplasmic vacuoles, the mice treated with 25 mg/kg flavone showed no or minimum vacuolation. In addition, the degeneration foci were greatly diminished and small number of mononuclear inflammatory cells were seen as illustrated in Figure 2D.

### 2.3. Markers of Oxidative Stress in Liver Tissue Homogenates

**Figure 3:**
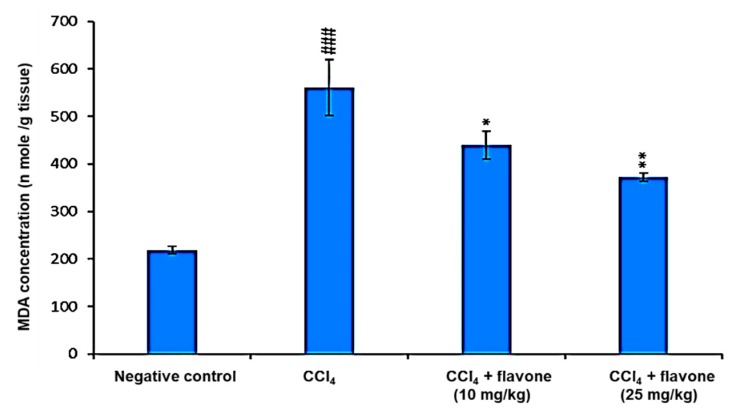
Effect of the flavone on MDA level in liver tissue homogenates. Data are presented as mean ± SD. Significantly (^###^ *p* < 0.001) different from normal control group. Significantly (* *p* < 0.05, ** *p* < 0.01) different from CCl_4_ model group. MDA: malonaldehyde.

MDA is the commonly used indicator for lipid peroxidation. Therefore, the levels of MDA in the liver homogenates were measured as in Figure 3. The levels of MDA in liver homogenates increased significantly (*p <* 0.001) in CCl_4_ model group compared to the control group. The treatment with flavone, especially high dose, exhibited a significant (*p* < 0.01) decrease in MDA production compared to CCl_4_ model group (Figure 3).

SOD acts as the first line of defense against superoxide anion. CAT, free radical scavenging enzyme, of oxygen-metabolizing cells that stop the potentially damaging reactivation of superoxide and hydrogen peroxide. GPx is an effective scavenger of cellular peroxides catalyzed by the oxidation of glutathione to glutathione disulfide. Consequently, the levels of SOD, CAT, and Gpx in the liver homogenates were measured. The results revealed that the administration of CCl_4_ significantly (*p* < 0.001) diminished the level of anti-oxidant enzymes in the liver tissue homogenate. Conversely, the flavone treatment, especially the high dose, induced a significant increase in SOD, CAT, and Gpx expression compared to the CCl_4_ model group (Table 1).

### 2.4. Apoptosis Regulators in Liver Tissue

**Figure 4:**
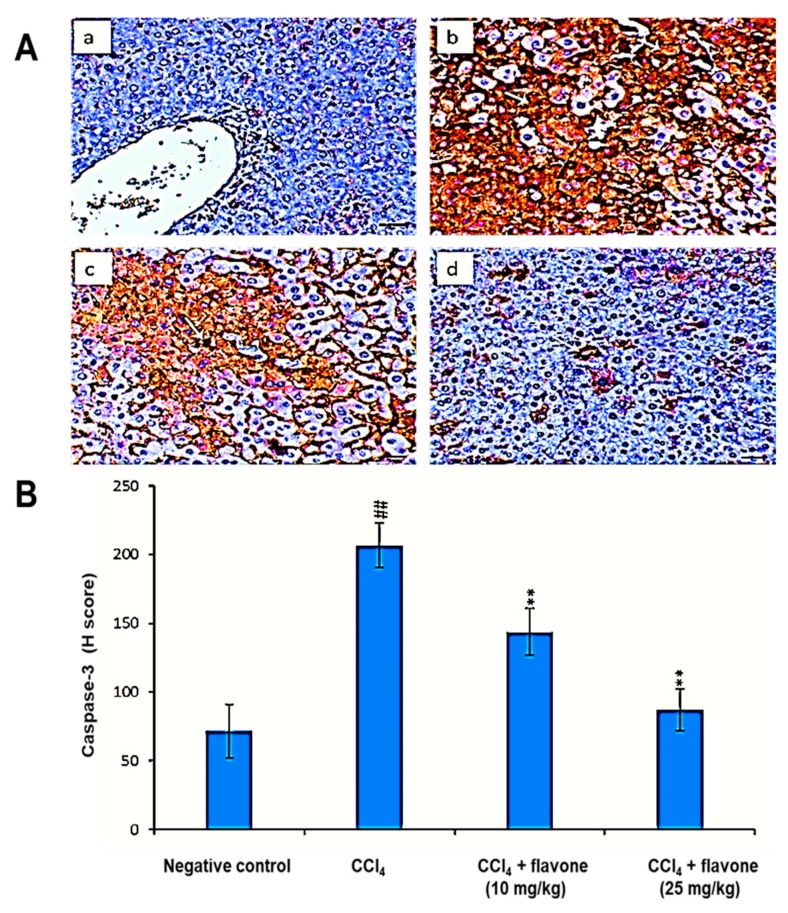
Effect of treatment with the flavone on the caspase-3 protein level in liver tissues. (**A**): Representative liver immunohistochemical graphs (a: control group, b: carbon tetrachloride (CCl_4_) group, c: CCl_4_ + flavone (10 mg/kg) group, d: CCl_4_ + flavone (25 mg/kg) group); (**a**) showed a noticeable expression of caspase-3 immunostaining in the liver tissues (arrow). (**b**) presented marked increase in caspase-3 expression within the liver tissues (arrow). (**c**) displayed mild to moderate decrease of caspase-3 expression in the liver tissues (arrow). (**d**) illustrated an obvious decrease in caspase-3 expression within the liver tissues (arrow). Scale bar = 100 µm. (**B**): Statistical analysis of caspase-3 H-score. Data are expressed as mean ± SD. Significantly (^##^ *p* < 0.01) different to negative control group. Significantly (** *p* < 0.01) different to the CCl_4_ group.

As displayed in Figure 4, the administration of CCl_4_ caused a significant (*p* < 0.01) elevation in caspase-3 levels compared to the control group. Treatment with flavone restored the protein levels of caspase-3 compared to CCl_4_ group. In particular, the high dose of the flavone (25 mg/kg) significantly (*p* < 0.01) decreased the level of caspase-3 to level near to the control group.

**Figure 5:**
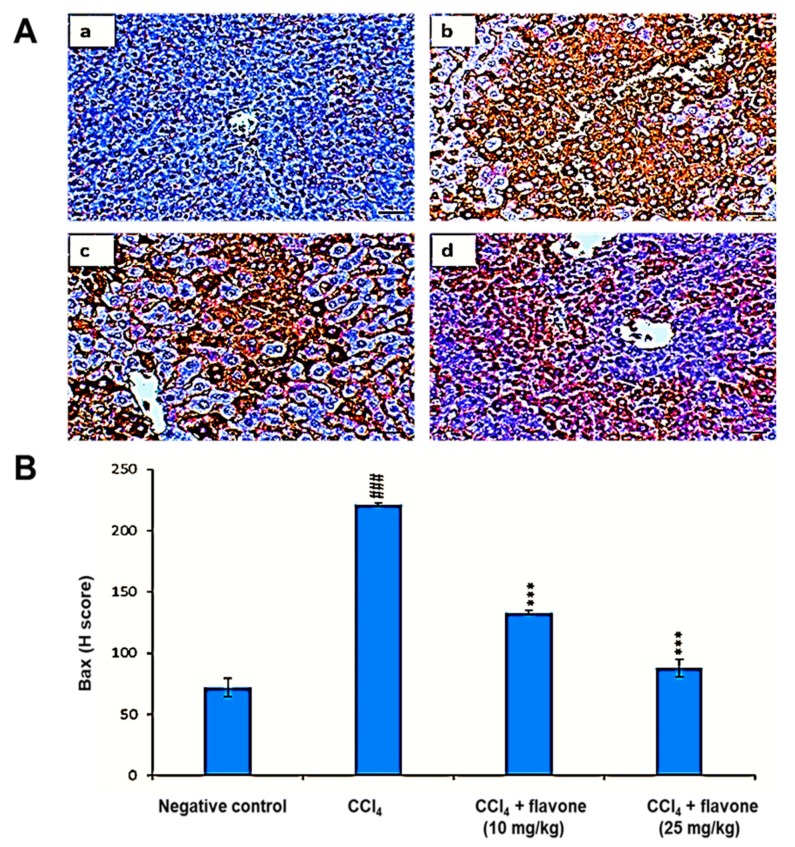
Effect of treatment with the flavone on the Bax protein level in liver tissues. (**A**): Representative liver immunohistochemical graphs (a: negative control group, b: carbon tetrachloride (CCl_4_) group, c: CCl_4_ + flavone (10 mg/kg) group, d: CCl_4_ + flavone (25 mg/kg) group); (**a**) showed a noticeable expression of Bax immunostaining in the liver tissues (arrow). (**b**) presented marked increase in Bax expression within the liver tissues (arrow). (**c**) displayed mild to moderate decrease of caspase-3 expression in the liver tissues (arrow). (**d**) illustrated an obvious decrease in Bax expression within the liver tissues (arrow). Scale bar = 100 µm. (**B**): Statistical analysis of Bax H-score. Data are expressed as mean ± SD. Significantly (^###^ *p* < 0.001) different to negative control group. Significantly (*** *p* < 0.001) different to the CCl_4_ group.

Compared to the levels of the control group, the Bax level was significantly (*p* < 0.001) increased in liver tissues after CCl_4_ injection. In contrast, Bax levels declined in mice injected with CCl_4_ and treated with flavone. A high dose of the flavone significantly (*p* < 0.001) decreased Bax levels, with an effect stronger than that observed in the other groups (Figure 5).

**Figure 6:**
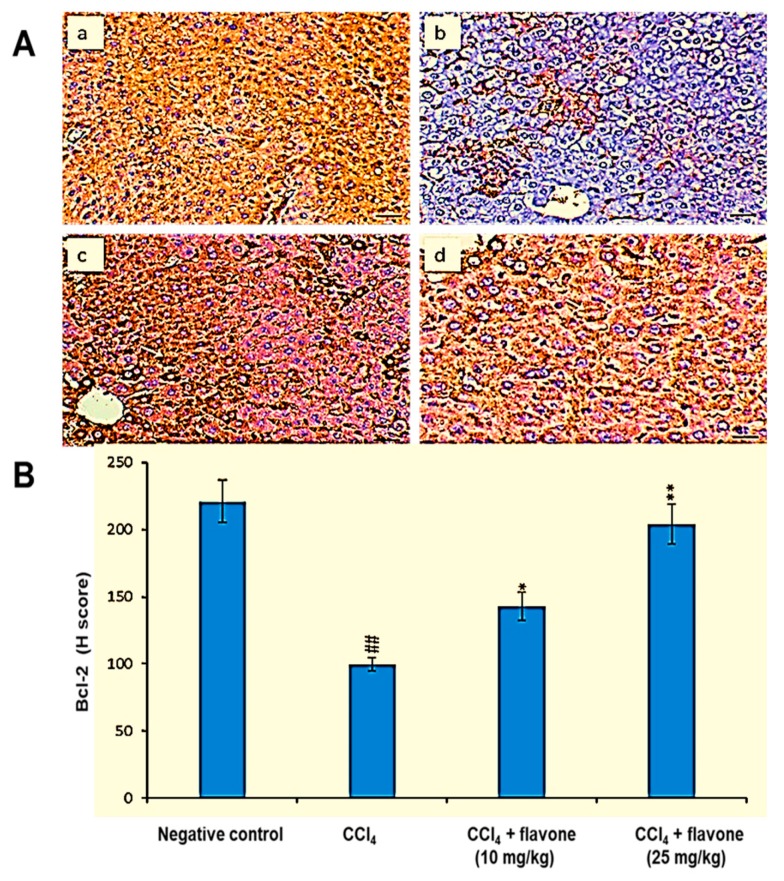
Effect of treatment with the flavone on the Bcl-2 protein level in liver tissues. (**A**): Representative liver immunohistochemical graphs (a: negative control group, b: carbon tetrachloride (CCl_4_) group, c: CCl_4_ + flavone (10 mg/kg) group, d: CCl_4_ + flavone (25 mg/kg) group); (**a**) showed a noticeable expression of bcl-2 immunostaining in the liver tissues (arrow). (**b**) presented marked decrease in Bcl-2 expression within the liver tissues (arrow). (**c**) displayed mild to moderate increase of Bcl-2 expression in the liver tissues (arrow). (**d**) illustrated an obvious increase in Bcl-2 expression within the liver tissues (arrow). Scale bar = 100 µm. (**B**): Statistical analysis of Bcl-2 H-score. Data are expressed as mean ± SD. Significantly (^##^ *p* < 0.01) different to negative control group. Significantly (* *p* < 0.05 and ** *p* < 0.01) different to the CCl_4_ group.

Figure 6 revealed that CCl_4_ significantly (*p* < 0.01) declined the expression level of Bcl-2 in liver tissues compared to those in the control group. Treatment with either low or high dose of the flavone can elevate the declined Bcl-2 levels. The flavone at concentration of 25 mg/kg significantly (*p* < 0.01) increased the declined expression of Bcl-2, with value more than that recorded in the other groups.

### 2.5. TGF-β1 Protein Levels in the Liver Tissue

**Figure 7:**
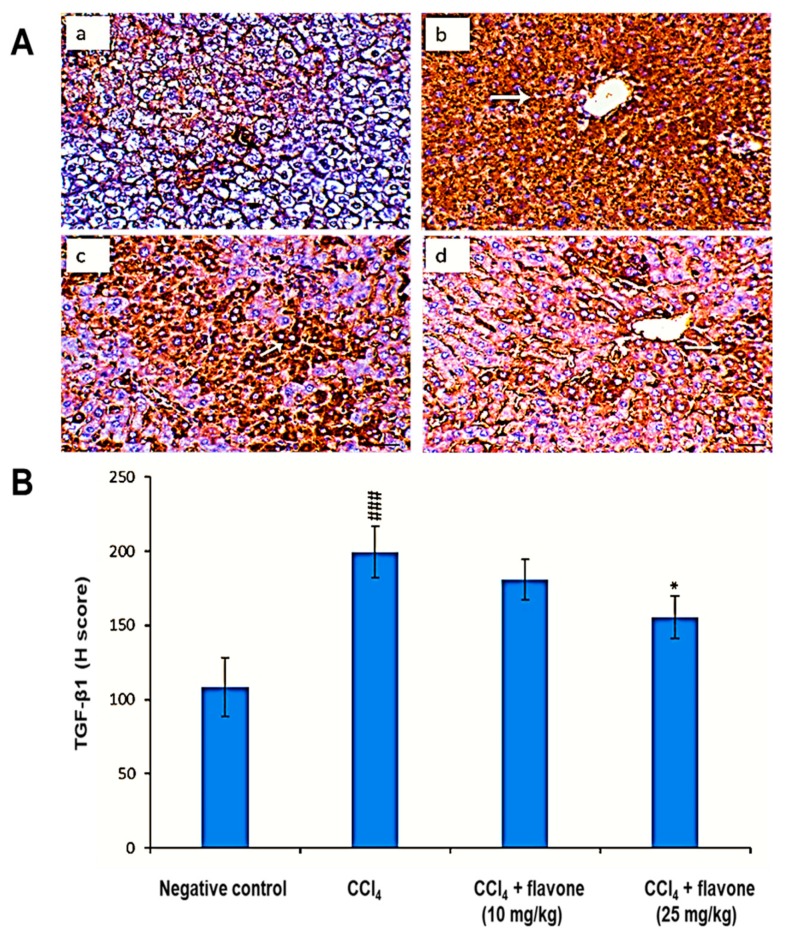
Effect of treatment with the flavone on the TGF-β1 protein level in liver tissues. (**A**): Representative liver immunohistochemical graphs (a: negative control group, b: carbon tetrachloride (CCl_4_) group, c: CCl_4_ + flavone (10 mg/kg) group, d: CCl_4_ + flavone (25 mg/kg) group); (**a**) showed a noticeable expression of TGF-β1 immunostaining in the liver tissues (arrow). (**b**) presented marked increase in TGF-β1expression within the liver tissues (arrow). (**c**) displayed mild to moderate decrease of TGF-β1 expression in the liver tissues (arrow). (**d**) illustrated an obvious decrease in TGF-β1 expression within the liver tissues (arrow). Scale bar = 100 µm. (**B**): Statistical analysis of TGF-β1 H-score. Data are expressed as mean ± SD. Significantly (^###^ *p* < 0.001) different to negative control group. Significantly (* *p* < 0.05) different to the CCl_4_ group.

As displayed in Figure 7, the administration of CCl_4_ caused a significant (*p* < 0.01) elevation in TGF-β1 levels compared to control group. Treatment with flavone restored the protein levels of TGF-β1. In particular, the high dose of flavone (25 mg/kg) significantly (*p* < 0.01) decreased the TGF-β1 level compared to the CCl_4_ model group.

### 2.6. CD31 Protein Levels in the Liver Tissue

**Figure 8:**
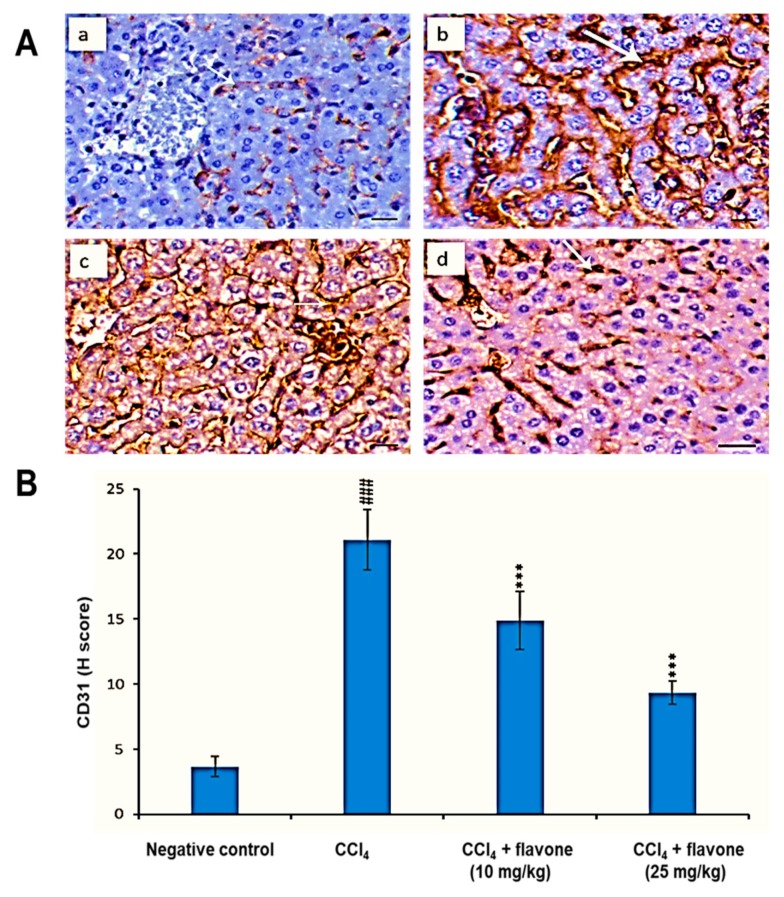
Effect of treatment with the flavone on the CD31 protein level in liver tissues. (**A**): Representative liver immunohistochemical graphs (a: negative control group, b: carbon tetrachloride (CCl_4_) group, c: CCl_4_ + flavone (10 mg/kg) group, d: CCl_4_ + flavone (25 mg/kg) group); (**a**) showed a noticeable expression of CD31 immunostaining in the liver tissues (arrow). (**b**) presented marked increase in CD31 expression within the liver tissues (arrow). (**c**) displayed mild to moderate decrease of CD31 expression in the liver tissues (arrow). (**d**) illustrated an obvious decrease in CD31 expression within the liver tissues (arrow). Scale bar = 100 µm. (**B**): Statistical analysis of CD31 H-score. Data are expressed as mean ± SD. Significantly (^###^ *p* < 0.001) different to negative control group. Significantly (*** *p* < 0.001) different to the CCl_4_ group.

Compared to the negative control group, the CD31 protein level was significantly (*p* < 0.001) augmented in liver tissues due to the action of CCl_4_ administration. In contrast, CD31 levels declined in CCl_4_ model control group after the treatment with the flavone. The high dose of the flavone significantly (*p* < 0.001) reduced the CD31 level, compared to that detected in the CCl_4_ group (Figure 8).

## 3. Discussion

Based on the fact that oxidative stress, apoptosis and angiogenesis mechanisms exhibited desirable effects for treatment of liver diseases in animal models, and to be clinically effective in preventing the disease progress or improving the outcome of patients [13,14,15,16]. Additionally, the in vivo model of CCl_4_-induced liver injury possesses a parallel molecular mechanism with chemical liver injury in humans [37]. Therefore, the current study aimed to investigate the in vivo protective effect of the flavone and its possible mechanisms in liver injury through studying its anti-antioxidant, anti-apoptotic, and anti-angiogenic activities in a mouse model of CCl_4_-induced liver injuries.

Hematoxylin and eosin (H&E) staining of the liver sections from CCl_4_ group showed multifocal granuloma like lesions within the whole hepatic parenchyma which consisted from mononuclear inflammatory cells infiltration mainly macrophage and lymphocytes mixed with multiple neoplastic cells. The outcome results were comparable to the previously reported histopathological examinations of liver injuries induced by CCl_4_ in mice [38,39,40]. On the other hand, compared to the CCl_4_ model group, hepatocytes were found to be normalized with minimum cellular necrosis after the flavone treatments, especially at 25 mg/kg, suggesting that the flavone was able to improve liver toxicity and histopathological changes produced by CCl_4_ in mice.

Lipid peroxide is a primary parameter which can be considered as a marker of oxidative injury, and hepatic MDA formation is commonly used as an indicator of liver tissue damage involving a series of chain reactions [41]. An increase in MDA levels in the liver suggested the enhancement of oxidative stress leading to tissue damage and failure of the antioxidant defense mechanisms to prevent the formation of excessive free radicals [42]. The results of the current study revealed marked increase in liver MDA levels in the liver tissue homogenate in CCl_4_-injected mice compared to the untreated normal mice. This result is consistent with previously reported studies [2,43,44,45]. On the other hand, the elevated hepatic MDA levels were significantly decreased by administration of the flavone. Therefore, we suggest that flavone exhibited protective effects against CCl_4_-induced liver damage in terms of preventing lipid peroxide formation and blocking oxidative chain reactions leading to decrease the lipid peroxidation and consequently reduce MDA levels in liver tissues.

Oxidative stress is produced due to the imbalance of cellular oxidation production and antioxidant capability. Therefore, it has become an attractive therapeutic strategy of antioxidant supplementation for reducing the risk of liver disease induced by superfluous free radicals [46,47]. SOD decrease the concentration of highly reactive superoxide radicals by transforming them to H_2_O_2_, while catalase (CAT), glutathione (GSH), and glutathione peroxidase (GPx) degrade H_2_O_2_ to protect the liver tissue from ROS produced by CCl_4_ exposure [48,49]. In our experiments, we found that administration of CCl_4_ to mice decreased the antioxidant capacity of mouse liver as evidenced by the declined levels of SOD, CAT and GPx, which were in agreement with the earlier results [50,51,52]. On the other hand, the treatment with the flavone significantly increased the reduced levels of antioxidant enzymes, especially at the high dosage of 25 mg/kg, suggesting that the flavone could protect liver against oxidative stress in CCl_4_-damaged mice via neutralizing the oxidative activity of CCl_4_-induced free radicals in the liver tissue.

Apoptosis is mediated by caspases upregulation, a class of cysteine proteins that cleave hundreds of various proteins. The antiapoptotic Bcl-2 protein inhibits apoptosis through the inhibition of pro-apoptosis protein BAX [53,54]. Our current results showed the decline of the Bcl-2 protein level in CCl_4_ model group compared to untreatd control group. Furthermore, in order to additionally understand the pathways of the anti-apoptotic effect of the flavone in CCl_4_-induced liver damage, caspase-3 and Bax protein levels were measured in the liver samples. Caspase-3 and Bax protein levels were significantly increased in the CCl_4_-injured liver tissues of mice compared to the untreated mice. Our findings were consistent with the previous studies where CCl_4_ increases Bax expression as well as caspase-3 regulation and reduces Bcl-2 protein expression demonstrating the induction of liver cell apoptosis [55,56]. On the other hand, the flavone exhibited antiapoptotic activity towards CCl_4_ induced apoptosis via the reduction of Bcl-2 levels and decline of the elevated levels of Bax as well as caspase-3.

TGF-β is an essential regulator of homeostasis in the liver tissue and its alteration attributes to liver damage and even hepatocellular carcinoma. Injured liver potentiates the increase of TGF-β1 levels participating in the cell death cascade [57]. Our results showed that the levels of TGF-β1 were significantly increased in the liver tissues of mice treated with CCl_4_. This finding is consistent with the previous studies reporting the increase of TGF-β1 in the serum of mice treated with CCl_4_ [58] and in hepatic tissue homogenate of acute liver injury [59]. Additionally, the regulation of the TGF-β1/Smad signaling pathway is a new strategy employed in the prevention of liver fibrosis, and plays vital roles during the processes of hepatocyte survival/restoration [60]. The flavone treatment markedly reduced the elevated TGF-β1 protein to a level similar to the untreated control group.

CD31 (also known as platelet-endothelial cell adhesion molecule-1, PECAM-1) is identified as a marker used for endothelial cells. It is expressed by the newly formed hepatic vasculature and its level reflected the condition of angiogenesis [61]. Additionally, CD31 is found and expressed on the surface of platelets and leukocytes and involved in endothelial cell junction, mediation of leukocyte transmigration between endothelial cells and binding of platelets to injured endothelium [62]. Moreover, CD31 participates in the removal of apoptotic leukocytes and aged neutrophils through promoting the tight binding of apoptotic cells with macrophage hence facilitate macrophage ingestion of dying cells [63].

Angiogenesis develops in parallel with liver injury and fibrogenesis [64,65,66]. To investigate the effect of the flavone on angiogenesis, we used CD31 as a marker for endothelial cells growth and development. Our immunohistochemical staining results showed an increased CD31 protein expression level in CCl_4_ model group compared to the control group. This was compatible with the literature where an increase in the expression levels of CD31 was noticed in mice injected with CCl_4_ [61,67]. The flavone decreased the elevated expression of CD31 and effectively attenuated liver injury induced by CCl_4_. Moreover, it has been stated that CD31 is promoting liver cell apoptosis [68], therefore, the ability of the flavone to inhibit hepatocytes apotosis and consequently reduce the over expression of CD31 is a promising finding.

Taken together, it was evidential from our findings that the treatment with the flavone can inhibit ROS formation, apoptosis and angiogenesis, secondary to hepatocytes damage in mice injected with CCl_4._ The process was explained by the observed inhibition of lipid peroxidation, activation of antioxidant enzymes, down-regulation of caspase-3 and Bax, increase the expression level of Bcl-2, and decrease the expression levels of TGF-β1 and CD31.

## 4. Materials and Methods

### 4.1. Extraction, Isolation and Elucidation of Flavone

The dried and ground plant material (1.8 kg) of aerial parts of Tamarix aphylla L. was extracted using a Soxhlet apparatus with 80% EtOH: H_2_O at 70–80 °C. The extract was concentrated in vacuo at 45 °C and freeze dried for 8 h to afford 300 g of a semi-solid dark material. The crude extract was suspended in distilled H_2_O and then successively partitioned between n-hexane, DCM, EtOAc, n-BuOH, and H_2_O to generate the corresponding sub-extracts. The DCM sub-extract (20 g) was loaded onto a normal-phase silica (NPS) gel column (70 × 5.6 cm) and eluted with gradients of MeOH: DCM. The compound, 5-dihydroxy-4′,7-dimethoxyflavone*,* was isolated from the DCM extract. The structure of the compound was established by chemical and spectral analysis, mainly MS (Nano ACQUITY UPLC, Uppsala, Sweden), UV (Shimadzu UV-160A, Tokyo, Japan) and ^1^H-NMR (Bruker DRX 400 spectrometer, Uppsala, Sweden). Recycling preparative HPLC (RP-HPLC (JAI LC-908W, Japan Analytical Industry Co. Ltd., Tokyo, Japan) with a YMC ODS H-80 or L-80 column (“YMC, Tokyo, Japan”) was used for the final purification of the flavone.

### 4.2. Animals

The protocol of the current study was approved by the Ethical Committee (26-8-2018) for Laboratory Animals of Science Faculty Menoufia University (No.: ECLA-SFMU-26818). A total of 32 adult Swiss albino male mice (7–8 weeks old) with an average body weight of 25 g were used in the current study. Mice were maintained in standard cages under controlled temperature conditions with a 12 h light/dark cycle and given food and water ad libitum.

### 4.3. Liver Injury Induction and Experimental Design

Liver injury was induced in mice by intraperitoneal (i.*p*.) administration of CCl_4_ (0.4 mL/kg) mixed in olive oil at ratio (1:4) twice a week for 6 consecutive weeks [69]. All mice groups were injected with CCl_4_ except group 1 (normal control); mice i.p. administrated olive oil twice a week for six consecutive weeks. After liver injury induction, mice were divided into three groups of 8 mice each as follows: Group 2 (CCl_4_ model group); mice injected with CCl_4_ and i.p. treated with olive oil. Group 3; mice injected with CCl_4_ and i.p. treated with the flavone at dose 10 mg/kg. Group 4; mice injected with CCl_4_ and i.p. treated with the flavone at dose 25 mg/kg. The treatment with the flavone was for twice a week for 4 consecutive weeks. Both doses (10 and 25 mg/kg) were selected according to a lethal dose study (data not shown). After the treatment period, mice were anaesthetized and sacrificed. Liver tissues were removed, and the left lobes of the liver tissues were immediately fixed in 10% neutral buffered formalin for histological and immunohistochemical examinations while the right lobes were used to prepare liver homogenates.

### 4.4. Histological Examination

The histological examination of liver was performed using 10% formaldehyde for fixation then liver tissues were embedded in paraffin wax and cut into sections with 5 μm thickness. Afterward, sections were caught with glass slide and stained with H&E. The slides were examined under a light microscope [70].

### 4.5. Estimation of Malondialdehyde (MDA) Level in Liver Homogenates

The 10% Liver homogenates were prepared with cold potassium phosphate buffer (50 mM, pH 7.4). The resulting suspension was centrifuged at 2000 rpm for 10 min. The supernatants were collected for further analysis. All treatments were done at 4 °C. The effect of flavone on MDA levels were determined as previously reported [34].

### 4.6. Determination of Anti-Oxidant Enzymes Levels in Liver Homogenates

The anti-oxidant activity of the flavone was determined through determination its effect on SOD, CAT and GPx levels in liver homogenates. The levels of the enzymes were determined by commercial kits according to the manufacturer’s instructions as previously reported [34].

### 4.7. Immunohistochemical Examination of Apoptosis and Angiogenesis-Related Protein Markers

The effect of the flavone on the protein expression levels of apoptosis-associated markers (Bax, Bcl-2, caspase-3) and angiogenesis-associated markers (CD31, and TGF-β1) was estimated using immunohistochemical assay as previously described [34]. In details, paraffin sections (5 μm) were dewaxed in xylene and rehydrated in graded alcohol. Antigen retrieval was achieved using microwave heating (20 min; 10 mmol/citrate buffer, pH 6). To block endogenous peroxidase activity, the sections were immersed in H_2_O_2_ (0.3%) for 30 min, followed by blocking with 5% non-fat dry milk for 30 min. The sections were incubated with primary monoclonal mouse anti-Bax, anti-Bcl-2, anti-Caspase-3, anti-CD31, and anti-TGF-β1 (BioGenex, Fremont, CA, USA) overnight at room temperature (RT) followed by three times washing with PBS (pH 7.4) for 5 min each. Sections were then incubated with a secondary horseradish peroxidase (HRP)-conjugated anti-mouse antibody for 30 min at RT and then developed by diaminobenzidine (DAB). The sections were stained with hematoxylin for 5 min. H-scores of Bax, Bcl-2, caspase-3, CD31, and TGF-β1were evaluated using a light microscope. H-score = ΣPi (i + 1), where i is the intensity of staining with a value of 1, 2 or 3 (weak, moderate or strong, respectively), and where Pi is the percentage of stained cells for each intensity, varying from 0 to 100%.

### 4.8. Statistical Analysis

The results were expressed as mean ± standard deviation (SD). The statistical significance was assessed by the Student’s t-test for comparisons of two groups, and one-way analysis of variance (ANOVA) followed by Tukey’s post-hoc test for multiple group comparisons, using GraphPad Prism 6 (GraphPad Software Inc., San Diego, CA, USA). A *p* value of < 0.05 was considered statistically significant.

## 5. Conclusions, Limitations and Future Research

The present study demonstrated that 3,5-dihydroxy-4′,7-dimethoxyflavone could be a helpful agent for the treatment of chemical-derived liver injury. The flavone exerted potentially protective antioxidant effects by increasing SOD, GPx, and CAT activities and decreasing lipid peroxidation. Also, the flavone exhibited anti-apoptotic effects by increasing Bcl-2 level and down-regulation of caspase-3 and Bax. Moreover, the flavone decreased the expression levels of TGF-β1 and CD31.

In despite of, this is the first investigation with clear evidence that the flavone may be a potential new complementary agent for the treatment of liver injury via the inhibition of ROS formation, apoptosis and angiogenesis. The current study has limitations about the beneficial anti-inflammatory activities of the flavone towards mouse model induced by CCl_4_. Therefore, future investigations are appreciated to more thoroughly reveal the effects of the flavone on different inflammatory cytokines. In particular, it would be of great interest to investigate whether the administration of flavone can represent an effective treatment against inflammation that induced in the mice livers due to the action of CCl_4_. Consequently, another study entitled “Differential cytokines expressions in liver tissue exposed to CCl_4_ in response to flavone from Tamarix aphylla” will cover the examination of the activity of the flavone on different inflammatory cytokines expressions such as TNF-α, IL-1α, IL-1β, IL-10 and IL-6.

## Figures and Tables

**Table 1:** Effect of the flavone on the levels of SOD, CAT, and Gpx in liver tissue homogenates.

Mice Group	SOD (U/g Tissue)	CAT (U/g Tissue)	Gpx (U/g Tissue)
Normal control	1.23 ± 0.03	2.69 ± 0.01	38.74 ± 3.61
CCl_4_ model group	0.32 ± 0.07 ^###^	1.98 ± 0.23 ^###^	21.76 ± 4.01 ^##^
Flavone (10 mg/kg)	0.58 ± 0.02^*^	2.27 ± 0.03^**^	27.65 ± 2.35^*^
Flavone (25 mg/kg)	0.89 ± 0.07^**^	2.48 ± 0.29^**^	30 ± 1.63^*^

Data are presented as mean ± SD. Significantly (^##^ *p* < 0.01, ^###^ *p* < 0.001) different from normal control group. Significantly (* *p* < 0.05, ** *p* < 0.01, *** *p* < 0.001) different from CCl_4_ model group. SOD: superoxide dismutase; CAT: catalase; Gpx: glutathione peroxidase.
